# Memory hierarchy characterization of SPEC CPU2006 and SPEC CPU2017 on the Intel Xeon Skylake-SP

**DOI:** 10.1371/journal.pone.0220135

**Published:** 2019-08-01

**Authors:** Agustín Navarro-Torres, Jesús Alastruey-Benedé, Pablo Ibáñez-Marín, Víctor Viñals-Yúfera

**Affiliations:** Departamento de Informática e Ingeniería de Sistemas - Aragón Institute for Engineering Research (I3A), Universidad de Zaragoza, Zaragoza, Spain; King Abdulaziz University, SAUDI ARABIA

## Abstract

SPEC CPU is one of the most common benchmark suites used in computer architecture research. CPU2017 has recently been released to replace CPU2006. In this paper we present a detailed evaluation of the memory hierarchy performance for both the CPU2006 and single-threaded CPU2017 benchmarks. The experiments were executed on an Intel Xeon Skylake-SP, which is the first Intel processor to implement a mostly non-inclusive last-level cache (LLC). We present a classification of the benchmarks according to their memory pressure and analyze the performance impact of different LLC sizes. We also test all the hardware prefetchers showing they improve performance in most of the benchmarks. After comprehensive experimentation, we can highlight the following conclusions: i) almost half of SPEC CPU benchmarks have very low miss ratios in the second and third level caches, even with small LLC sizes and without hardware prefetching, ii) overall, the SPEC CPU2017 benchmarks demand even less memory hierarchy resources than the SPEC CPU2006 ones, iii) hardware prefetching is very effective in reducing LLC misses for most benchmarks, even with the smallest LLC size, and iv) from the memory hierarchy standpoint the methodologies commonly used to select benchmarks or simulation points do not guarantee representative workloads.

## Introduction

Much of the experimental research in computer architecture is based on feeding a simulator or a real machine with benchmarks that are representative of current or future software in a certain application field. Hence, diverse corporations (e.g. SPEC [1]), research groups (e.g. CloudSuite [2]), communities (e.g. TACLeBench [3]) and even certain companies (e.g. EEMBC [4]) propose benchmark suites composed of a number of applications focused on specific fields such as general purpose computing, cloud computing, real time or embedded processing, respectively.

According to SPEC Corporation, SPEC CPU2017 contains a collection of next-generation and industry-standardized benchmarks aimed at stressing the processor, memory subsystem and compiler [5].

Benchmark characterization is one of the first tasks carried out by the computer architecture community. Among its goals, we can highlight the selection of samples for simulation, the classification of benchmarks according to certain characteristics, or the detection of non-optimal run-time behaviors.

Implementing a new hardware concept in a real system is unfeasible in most cases, due to its high cost or the impossibility of its subsequent modification. An alternative is to use a simulator which models at the desired level of detail (e‥g. cycle-level) the behavior of a complex system such as a multicore processor with a multi-level memory hierarchy and an interconnection network. However, the complete execution of a benchmark in these simulators may require months or even years. Thus, sampling techniques are used to identify small sections of a benchmark that approximate the behavior of the full application [6, 7].

Benchmarks with certain characteristics are selected to evaluate the performance of new proposals. For example, research on shared cache replacement algorithms frequently selects a mix of benchmarks with different degrees of pressure on the memory hierarchy: some of them show high cache utilization while others do the opposite [8, 9].

In this paper, we characterize the interaction of the SPEC CPU2006 and CPU2017 suites with the Intel Xeon SP’s memory hierarchy. The analysis of CPU2017 is of special interest since it is a recent suite and there has been little research on it [10]. Regarding the processor, it also brings relevance to this study because the Intel Xeon SP family has been released in July 2017 and incorporates significant changes in the memory hierarchy: the private L2 cache size has quadrupled and the shared LLC, unlike all previous Intel processors, has been designed following a mostly non-inclusive policy. AMD chose similar policies since its inception, namely strict exclusion between the private cache levels, and mostly-exclusion between private cache levels and the LLC. So we think non-inclusive content policies seem to be a consolidating trend worth focusing. Finally, we can point out here that a similar study on inclusive policies could lead to an interesting performance comparison of both memory subsystems. However, such a study is outside the scope of this paper.

The contributions of this paper are:

- A characterization of the benchmarks’ sensitivity to LLC capacity and hardware prefetching.
- An evaluation of the impact of the different hardware prefetchers present in the Intel Xeon Skylake-SP [11] on the average count of cycles per instruction (CPI) and the main memory bandwidth.
- A characterization of the temporal evolution of the benchmarks aimed at identifying relevant sections for simulation.

**Fig 1:**
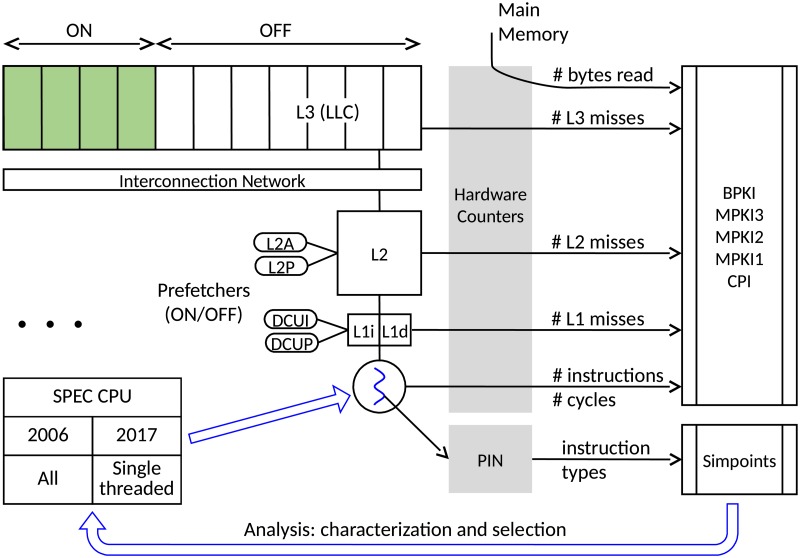
Outline of the methodology.

In Fig 1 we outline the relevant hardware components and raw measures we use in our methodology. For instance, we compute misses per kilo instruction in the first, second and last-level caches (MPKI1/MPKI2/MPKI3) and CPI (average number of cycles per instruction).

Most previous work uses simulators to obtain hardware events (executed instructions, memory references, cache misses …) and metrics (cache miss ratio, prefetch accuracy …) for different LLC sizes, which imposes the use of sampling and precludes running the full benchmarks until completion. Besides, processor details as commercial hardware prefetchers may not be accurately modelled due to a lack of information about their behavior. Unlike that methodology, we collect events with the hardware counters provided by Intel processors [12]. We use *Intel Resource Director* [13] to limit the number of LLC ways allocated to an application. The behavior of the different hardware prefetchers is studied by turning them on selectively by using the corresponding control register. In this way, we are able to combine full execution with both LLC size variation and individual hardware prefetch analysis.

Moreover, we also resort to the instrumentation tool PIN to analyze instruction operation codes [14], and feed them into the simpoint package to obtain representative simulation intervals according to that proposal [7].

This paper is organized as follows: section *State of the art* introduces the state of the art about characterization methodology and selection of simulation intervals. Section *Methodology* describes the execution environment, benchmarks and metrics used. Experimental results are shown in section *Evaluation*. Finally, in section *Conclusions* we summarize the paper contributions.

## State of the art

The characterization of a new benchmark suite is a recurrent research activity in the computer architecture community. In this section, we present the state of the art in two areas: benchmark characterization and selection of simulation intervals.

### Benchmark characterization methodologies

Benchmark characterization may be carried out through simulation or by using hardware counters. On the one hand, simulation provides a flexible experimentation framework that allows the evaluation of different memory hierarchy configurations, such as cache sizes or replacement policies. Unfortunately, hardware components in recent processors, such as prefetchers, can not be accurately modelled since their implementation details are not fully disclosed. Moreover, a complete benchmark simulation may require weeks or months, so studies based on simulation typically characterize only a small section of the selected benchmarks.

On the other hand, real execution is able to capture the behavior of state-of-the-art hardware components. Performance monitoring support included in commercial systems collects execution events that can be used to obtain metrics that characterize benchmark behavior during their real execution with a very low overhead. However, using real execution makes it difficult to perform design space exploration, since hardware configuration capabilities are limited.

There are many papers devoted to the SPEC CPU2006 characterization. For instance, Jaleel et al. characterize the behavior of the suite with different cache sizes on a simulator [15], Korn et al. study its performance according to page size [16], and Bird et al. analyze its performance by running on an Intel Core2Duo [17].

Regarding SPEC CPU2017, we have only found two characterization studies: Limaye et al. [18] and Panda et al. [10]. They analyze the behavior of benchmarks on an Intel processor from the Haswell family. Hardware counters are used to collect the amount and type of executed instructions, memory footprint, and cache misses at all levels of the memory hierarchy. Both papers end up presenting a methodology to classify benchmarks. Regarding the characterization of the use of the memory hierarchy, they show some limitations. Namely, characterization is performed on old systems, local miss ratios are used as performance metric instead of MPKI, and the sensitivity to cache size or hardware prefetching is not studied.

With respect to the content management in shared LLCs, many processors use an inclusive policy (LLC content is a superset of all private caches), however, using instead a mostly non-inclusive policy (LLC acts as a victim cache which may, or may not, evict cache lines on hits) seems to gain momentum through more elaborated coherence protocols. AMD calls the same policy “mostly-exclusive”, and started using it in its first processor with shared LLC, the 2007 4-core Opteron Barcelona [19]. All the following AMD processors, such as the 6-core Istanbul (2009), the 12-core Magny Cours (2010), the 16-core Bulldozer (2011) or, recently the 4-core Zen Core Complex (2016) evolved in cache sizes, coherency protocols and core features, but all have maintained the same mostly-exclusive contents policy. So we think characterizing benchmarks through the Intel Skylake-SP fits well with this trend.

In this work, hardware counters have been used to obtain metrics about the execution of the benchmarks on a Skylake-SP processor. Intel’s Model Specific Registers (MSR) allow us to independently enable or disable the different hardware prefetchers. The *Intel Cache Allocation Technology (CAT)* allows us to vary the LLC space occupied by an application modifying its number of allocated ways. In this way, we characterize the behavior of the entire benchmark with different hardware configurations of the memory hierarchy.

### Selection of benchmarks and simulation intervals

SPEC CPU2006 and CPU2017 are composed of several applications, some of them with different inputs, resulting in multiple application-input combinations. We define *benchmark* as an application-input pair. For example, there are 29 applications and 55 benchmarks (application-input pairs) in the CPU2006 suite. The execution time of a complete benchmark on a simulator may last weeks or even months, which makes the simulation of a suite unfeasible. To reduce this time, a two-level sampling can be carried out. First, a subset of benchmarks is selected. Second, one or more fragments of the complete execution representing the overall behavior are chosen as simulation intervals. In the literature, several successful sampling techniques have been proposed, such as *Hierarchical Clustering* [18] for benchmark selection, and *SimFlex* [6] or *SimPoint* [7] for intervals selection.

#### Hierarchical Clustering [18]

The *Hierarchical Clustering* methodology is applied in three steps: i) execution of all benchmarks to obtain 20 metrics through hardware counters, ii) analysis of the main components to reduce the number of metrics to 4, and iii) clustering of similar benchmarks.

In the first step, the authors select microarchitecture-independent metrics which are related to the type of instructions executed and their proportions. Therefore, this methodology does not consider the behavior of the memory hierarchy as a parameter to guide sampling.

#### SimFlex [6]

The *SimFlex* methodology uses statistical sampling theory to select simulation intervals. It identifies numerous small-sized intervals that are distributed throughout all the application execution, ensuring that they are representative of the benchmark.

However, *SimFlex* has an important drawback when it is applied to cache memory hierarchy research: the simulation intervals do not have enough extension to provide accurate data without a previous cache warm-up. Warming memory structures has an unacceptable overhead when simulating large caches or a large number of intervals.

#### SimPoint [7]

*SimPoint* is one of the most used methodologies to select simulation intervals. First, it splits up the execution of a benchmark into intervals of equal number of instructions. For each interval, *Simpoint* calculates a signature that contains the number of executions of each basic block. Then, *SimPoint* executes the *k-Means* algorithm to group different intervals into clusters called phases. The intervals of a given phase execute similar code and therefore are expected to exhibit a similar behavior in the system (misses in the memory hierarchy, CPI …). Finally, the centroid is selected as the most representative interval of each phase.

Although *SimPoint* offers several simulation intervals for each benchmark, most research related to memory hierarchy design uses only the most representative interval.

One of the contributions of this paper is to assess the representativeness of the intervals selected by *SimPoint* regarding the interaction with the memory hierarchy. Towards that end we analyze the temporal evolution of different metrics, namely CPI, MPKI2 and MPKI3, across the whole application execution. By plotting such metrics as a function of time and superposing the first three intervals selected by *SimPoint*, we will see how well they match the memory hierarchy dynamics.

## Methodology

### Runtime environment

The SPEC CPU2006 and the single-threaded CPU2017 benchmarks have been executed on a system with an *Intel Xeon Gold 5120* processor, code-named Skylake-SP (Skylake Scalable Performance, SKL-SP for short), and 96 GiB of DRAM running a CentOS 7 Linux with the 3.10 kernel. The system specifications are shown in Table 1. The processor integrates 14 cores. Each core contains a split first level cache (32 KiB for instructions and 32 KiB for data), a 1 MiB unified second level cache with 16 ways, and four hardware prefetchers. All cores share a 19.25 MiB LLC with 11 ways.

**Table 1:** System specifications.

Processor	Intel Xeon Gold 5120 (Skylake-SP)
Main Memory	96 GiB DDR4
L1 I-Cache	32 KiB, 64 B line size, 8 ways
L1 D-Cache	32 KiB, 64 B line size, 8 ways
L2	1 MiB, 64 B line size, 16 ways
L3	19.25 MiB, 64 B line size, 11 ways
OS	CentOS 7, kernel: 3.10

In order to assure reproducibility, each benchmark has been executed alone and pinned to a given core. All measures of hardware events have been taken from hardware counters driven by *Perf*, a Linux profiler tool [20].

We use *Intel Cache Allocation Technology* (CAT) [21], a tool included in the *Intel Resource Director*, to modify the amount of LLC storage allocated to an application during its execution. Besides, we selectively enable or disable the hardware prefetchers through the 0x1A4 *Model Specific Register* (MSR). This procedure allows us to characterize the behavior of an entire benchmark on a real system for different hardware prefetching configurations and LLC sizes.

### Intel SKL-SP memory hierarchy

The SKL-SP is the first Intel processor family that uses a mostly non-inclusive memory hierarchy. In all of its previous cache organizations, Intel used instead the inclusion policy, which enforces that all the private caches (L1 and L2) content of all the cores are also stored in the shared LLC. Inclusion leads to designs with relatively small private caches and a large shared LLC. By contrast, in non-inclusive hierarchies, the LLC content is largely independent of the private caches content. Changing the policy from inclusive to mostly non-inclusive may have allowed Intel to redistribute cache chip area, by enlarging private caches (from 256 KiB to 1 MiB) and diminishing LLC (from 2.5 MiB to 1.375 MiB per core). However, since now LLC stores less replicated content, reducing its size does not necessarily imply lowering its effective capacity. According to D. Kanter, *the new cache design reduces the processor’s L2 miss rate by about 40% on average for the SPECint_rate2006 suite, whereas the L3 miss rate barely increases* [11].

### Benchmarks

The CPU2006 and CPU2017 suites have been compiled following the official documentation provided by SPEC. CPU2006 has been compiled with gcc 4.9.2 and the options -O3 -fno-strict-aliasing. CPU2017 has been compiled with gcc 6.3.1 and the base flags. -DBIG_MEMORY has been used for deepsjeng and -m64 when required.

The CPU2017 speed versions of application xz and all the floating point (fp) applications are multi-threaded. We do not consider them in our study because the characterization of multi-threaded benchmarks requires a very different methodology. Therefore, all CPU2006 and single-threaded CPU2017 applications have been executed with all the so called “reference” inputs (one or more input data sets representative of real behavior, different from the “train” or “test” input data sets not devised for measurement purposes). As said before, each application-input pair constitutes a benchmark.

Table 2 shows the 106 benchmarks tested across the two suites, from a total of 43 applications, 17 integer and 26 floating point. Some applications appear only in one suite, as astar (SPEC CPU2006 int) or blender (SPEC CPU2017 fp), while some others have evolved and are in both suites, as gcc. Moreover, some CPU2017 applications have both speed and rate versions. For instance, the integer application mcf has one CPU2006 version and two CPU2017 versions, one to produce the SPECrate metric (_r), and the other one the SPECspeed metric (_s). For each benchmark, the table specifies an input identifier (#), the input name (Input) and the measured instruction count (Inst.).

**Table 2:** Benchmarks tested, divided between integer (int column) and floating point (fp column). Filled cells in columns “2006” and “2017” mean the benchmark appears in the corresponding suite. The columns labeled “_r” and “_s” refer to the application versions producing SPECrate and SPECspeed metrics, respectively. Columns “Inst.” and “#” show instruction count (x10^12^) and input identifier, respectively.

2006			int	2017				2006			fp	2017		
*Inst*.	*Input*	*#*	*Name*	*#*	*Input*	*Inst*.		*Inst*.	*Input*	*#*	*Name*	*#*	*Input*	*Inst*.
						_r	_s							_r
0.36	BigLakes	1	**astar**								**blender**	1	sh3	1.73
0.75	rivers	2						2.56	*None*	1	**bwaves**	1	bwaves_1	1.30
0.41	source	1	**bzip2**									2	bwaves_2	1.57
0.17	chicken.jpg	2										3	bwaves_3	1.40
0.29	liberty.jpg	3										4	bwaves_4	1.83
0.53	program	4									**cactuBSSN**	1	spec_ref	1.11
0.58	text.html	5						2.73	benchADM	1	**cactusADM**			
0.33	combined	6						4.25	hypervis	1	**calculix**			
			**deepsjeng**	1	ref	1.87	2.18				**cam4**	1	*None*	2.69
			**exchange2**	1	6	2.91	2.91	1.65	23	1	**dealII**			
0.07	166	1	**gcc**	1	pp.c -O3	0.20					**fotonik3d**	1	*None*	1.95
0.14	200.00	2		2	pp.c -O2	0.23		0.11	cytosine	1	**gamess**			
0.12	c-typeck	3		3	small.c -O3	0.23		0.09	h2ocu2+	2				
0.09	cp-decl	4		4	ref32.c -O5	0.19		0.37	triazolium	3				
0.10	expr	5		5	ref32.c -O3	0.26		1.72	*None*	1	**gemsFDTD**			
0.14	expr2	6		1	-fipa-pta		1.21	1.95	gromacs	1	**gromacs**			
0.17	g23	7		2	-fin = 1000		0.52				**imagick**	1	refrate	4.59
0.15	s04	8		3	-fin = 24000		0.50	1.24	reference	1	**lbm**	1	reference	1.28
0.05	scilab	9						0.18	leslie3d	1	**leslie3d**			
0.02	13x13	1	**gobmk**					0.10	su3imp	1	**milc**			
0.06	nngs	2									**nab**	1	1am0	2.09
0.03	score2	3						2.28	namd	1	**namd**	1	apoa1	1.78
0.02	trevorc	4									**parest**	1	ref	3.39
0.03	trevord	5						0.94	SPEC-ref	1	**povray**	1	SPEC-ref	3.31
0.50	baseline	1	**h264ref**								**roms**	1	ocean2	2.71
0.32	main	2						0.34	pds-50	1	**soplex**			
2.89	sss_main	3						0.35	ref	2				
0.86	nph3	1	**hmmer**					3.39	ctlfile	1	**sphinx3**			
1.82	retro	2						3.44	*None*	1	**tonto**			
			**leela**	1	ref	2.11	2.11	2.93	*None*	1	**wrf**	1	*None*	4.20
1.65	1397	1	**libquantum**					1.92	*None*	1	**zeusmp**			
0.32	inp	1	**mcf**	1	inp	0.92	1.65							
0.54	omnetpp	1	**omnetpp**	1	General	1.09	1.06							
1.05	checkspam	1	**perlbench**	1	checkspam	1.22	1.22							
0.36	diffmail	2		2	diffmail	0.70	0.70							
0.66	splitmail	3		3	splitmail	0.67	0.67							
2.26	ref	1	**sjeng**											
			**x264**	1	-pass 1	0.52	0.52							
				2	-pass 2	1.96	1.96							
				3	-seek 500	1.99	1.99							
0.99	t5	1	**xalancbmk**	1	t5	1.27	1.27							
			**xz**	1	cld	0.40								
				2	cpu2006	1.04								
				3	combined	0.57								
**Total**						**Total**	**Total**	**Total**						**Total**
18.84						20.34	20.46	32.51						36.91
**AVG**						**AVG**	**AVG**	**AVG**						**AVG**
0.54						1.02	1.36	1.63						2.31

As a general rule, we can see a significant increase of individual instruction counts in the CPU2017 benchmarks with respect to the CPU2006 ones, even though if we take into account the aggregate figures the difference flattens.

### Metrics

When measuring performance of an application running on a system, the ultimate metric is execution time. Any other metric is intended to help understand why an execution time is obtained or to show the influence of some subsystem on it. Therefore, a performance metric is only useful if it gives insight into execution time variations. Since we are interested in characterizing the memory hierarchy as a performance enabler or limiter, we measure miss ratios and memory bandwidth consumption in addition to execution time. Specifically, we consider number of cycles per instruction (CPI), number of misses per thousand instructions in the different cache levels (misses per kilo instruction, MPKI), and number of bytes read from main memory per thousand instructions (bytes per kilo instruction, BPKI), as performance metrics.

Previous works often use the LLC local miss ratio (#LLC misses / #LLC accesses) instead of MPKI [18]. However, the LLC local miss ratio does not consider how often the LLC is accessed and thus, it does not correlate well with execution time. For instance, a large reduction in the local LLC miss ratio may not decrease the execution time if the average number of LLC accesses per instruction is very low. Conversely, a slight LLC local miss ratio reduction can significantly reduce the execution time if the number of LLC accesses per instruction is high. On the other hand, MPKI is a metric that correlates much better with execution time. MPKI is a global metric, since it is relative to the number of instructions executed. Few misses per instruction imply little penalty in time and vice versa.

With respect to memory bandwidth consumption, we use BPKI instead of bytes per time (i.e. bytes per kilo cycle, BPKC) because we want to quantify prefetching overhead. As we will see in the results section, prefetching usually results in a significant decrease in the execution time, which in turns causes a BPKC increment, even when the number of bytes read from memory is not increased. BPKI, on the other hand, measures bandwidth consumption per unit of work performed. The value of BPKI in the system without prefetching can be considered a minimum. Any increase in this metric when prefetching is enabled indicates a waste of the memory bandwidth resource.

## Evaluation

### Identification of memory intensive benchmarks

All the SPEC CPU2006 benchmarks (55 from 29 applications) and the CPU2017 single-threaded benchmarks (51 from 23 applications) have been executed first with limited memory resources, disabling all hardware prefetchers and using the minimum LLC size available, 1.75 MiB, resulting from enabling only one of the eleven LLC ways.

**Fig 2:**
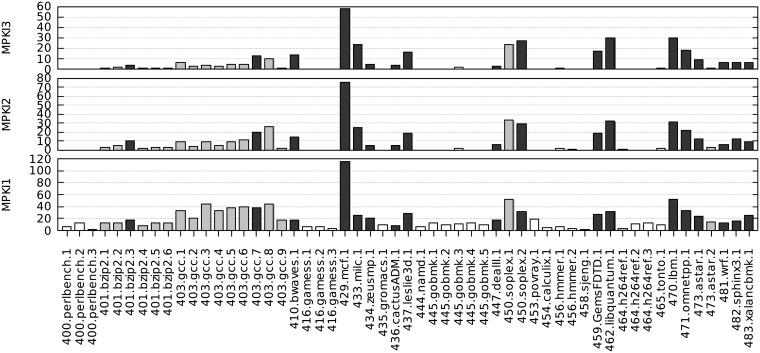
MPKI1, MPKI2 and MPKI3 for all SPEC CPU2006 benchmarks, sorted by benchmark number.

**Fig 3:**
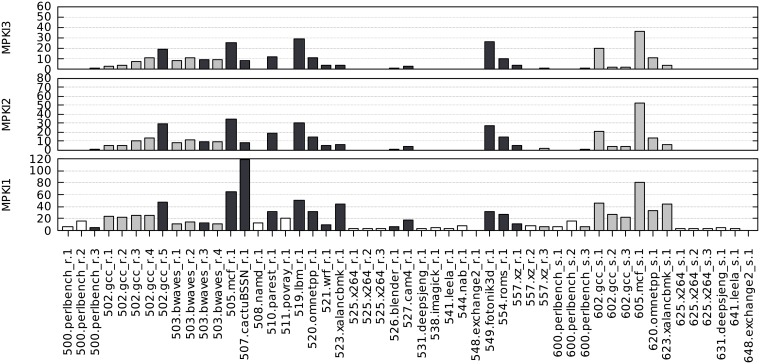
MPKI1, MPKI2 and MPKI3 for all SPEC CPU2017 single-threaded benchmarks, sorted by benchmark number.

For each benchmark, we have measured its miss ratios in the three data cache levels (MPKI1, MPKI2, MPKI3). These metrics are shown in Figs 2 and 3 for SPEC CPU2006 and CPU2017, respectively.

If we select for each application the benchmark with the highest MPKI3, the average value of MPKI1 is similar for CPU2006 and CPU2017 (21.9 and 21.8, respectively). However, the average values of MPKI2 and MPKI3 are clearly higher in CPU2006 (12.4 and 10.2) than in CPU2017 (8.1 and 6.6). Therefore, a first conclusion is that SPEC CPU2017 does not put more pressure on the memory hierarchy, rather the opposite.

In order to select a set of memory-intensive benchmarks we proceed as follows. Firstly, we identify benchmarks with very low MPKI2 and MPKI3 ratios, namely (both below 1.0). Under these circumstances the SKL-SP private caches are sufficient to meet the storage needs and we assume that the LLC behavior has no interest. Thus, we propose to leave out from further analysis the 43 benchmarks indicated by white-filled bars in the MPKI1 axes (22 out of 55 in CPU2006, and 21 out of 51 in CPU2017). Secondly, for all the remaining benchmarks we select a single representative for each application, the one with the highest MPKI2-3 miss ratios (black-filled bars in the figure). Notice that in some cases, the specific application selection extends to both CPU2006 and 2017, as is the case, for example, of mcf. Table 3 shows the resulting 33 benchmarks (18 from CPU2006 and 15 from CPU2017) that form our selected workload of memory-intensive benchmarks which will be analyzed in depth in the next sections.

**Table 3:** Selected benchmarks and their performance metrics for minimum LLC size and no prefetching.

2006					2017				
*Benchmark*	*MPKI1*	*MPKI2*	*MPKI3*	*CPI*	*Benchmark*	*MPKI1*	*MPKI2*	*MPKI3*	*CPI*
401.bzip2.3	18.4	10.2	4.5	0.88	500.perlbench_r.3	5.8	1.9	1.5	0.71
403.gcc.7	38.1	20.6	13.0	1.99	502.gcc_r.5	47.2	29.3	19.1	2.16
410.bwaves.1	17.5	14.8	14.6	0.96	503.bwaves_r.3	12.5	9.9	9.7	0.75
429.mcf.1	116.3	75.9	59.0	5.08	505.mcf_r.1	65.8	34.2	25.7	1.97
433.milc.1	26.2	25.1	24.4	1.92	507.cactuBSSN_r.1	118.3	8.8	8.4	1.02
434.zeusmp.1	21.5	5.4	5.0	0.78	510.parest_r.1	31.5	18.7	12.5	1.34
436.cactusADM.1	8.0	5.1	4.5	0.91	519.lbm_r.1	50.9	30.7	29.0	1.59
437.leslie3d.1	29.0	18.3	17.0	1.16	520.omnetpp_r.1	32.3	14.1	11.3	1.84
447.dealII.1	18.7	6.6	3.8	0.72	521.wrf_r.1	10.9	5.6	4.7	1.26
450.soplex.2	31.9	29.1	27.6	2.35	523.xalancbmk_r.1	44.2	6.7	4.4	1.11
459.GemsFDTD.1	27.1	18.8	17.5	1.39	526.blender_r.1	7.5	1.9	1.5	0.68
462.libquantum.1	32.8	32.2	30.5	1.22	527.cam4_r.1	18.5	4.4	3.0	0.73
470.lbm.1	52.3	31.6	30.2	1.42	549.fotonik3d_r.1	32.3	27.8	27.0	2.06
471.omnetpp.1	34.6	22.9	18.4	1.52	554.roms_r.1	27.6	14.4	10.9	1.07
473.astar.1	24.8	12.4	9.8	1.08	557.xz_r.1	11.9	5.4	4.3	1.34
481.wrf.1	12.2	6.4	6.0	0.82					
482.sphinx3.1	16.3	12.4	6.9	0.68					
483.xalancbmk.1	26.3	9.1	6.1	0.64					

### Sensitivity to LLC size and hardware prefetching

In this experiment, we study the sensitivity of the memory intensive benchmarks to LLC size and hardware prefetching. The benchmarks selected in the previous section have been executed with five LLC sizes. For each LLC size, two executions have been performed: all prefetchers enabled and all disabled.

The evaluated LLC sizes are: 1.75 MiB, 3.5 MiB, 7 MiB, 14 MiB and 19.25 MiB, corresponding to associativities of 1, 2, 4, 8 and 11, respectively.

**Fig 4:**
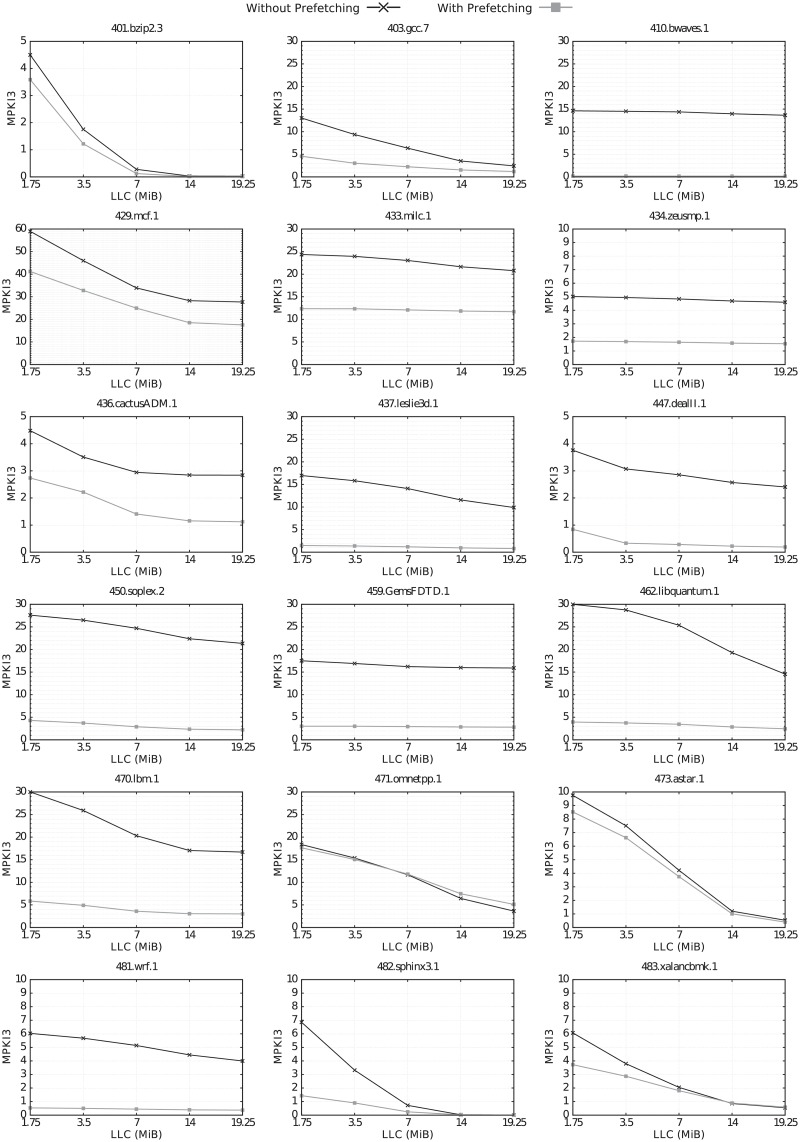
MPKI3 vs. LLC size for the selected CPU2006 benchmarks, with and without prefetching.

**Fig 5:**
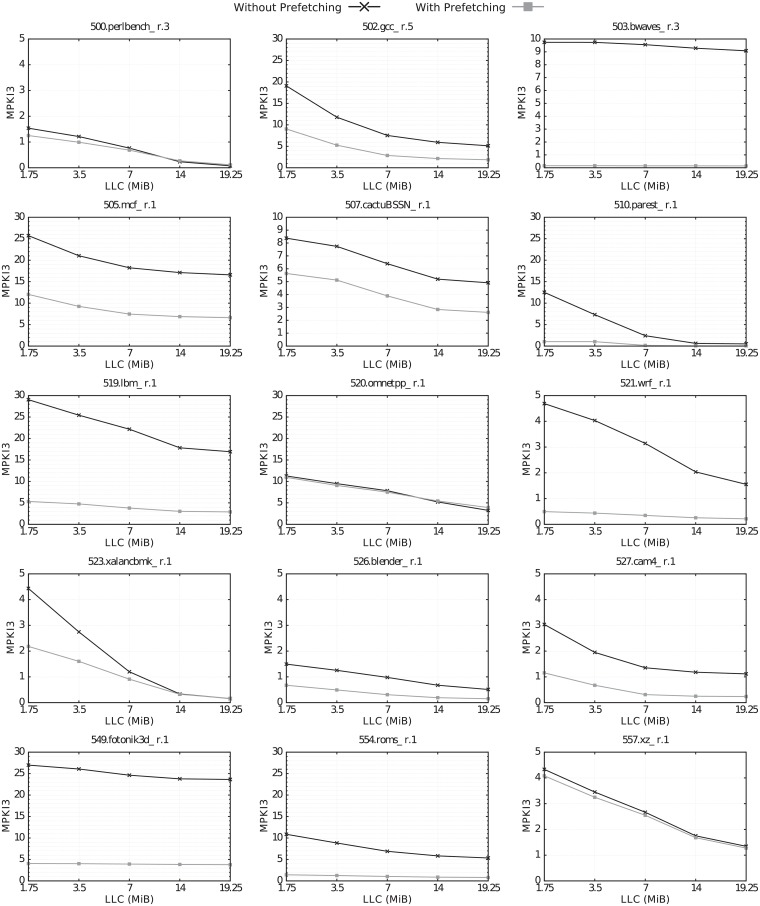
MPKI3 vs. LLC size for the selected CPU2017 benchmarks, with and without prefetching.

Figs 4 and 5 show the MPKI3 of the selected benchmarks for the different LLC sizes, with and without hardware prefetching.

Without hardware prefetching, increasing the LLC size results in an MPKI3 reduction for almost all benchmarks of both suites, with the exception of 410.bwaves, 434.zeusmp, and 459.GemsFDTD in CPU2006, and 503.bwaves in CPU2017.

With hardware prefetching, the MPKI3 improvement achieved by increasing the LLC size is considerably reduced for 6 CPU2006 benchmarks (433.milc, 437.leslie3d, 447.dealII, 450.soplex.2, 462.libquantum and 481.wrf), and for 5 CPU2017 benchmarks (510.parest, 519.lbm, 521.wrf, 549.fotonik3d and 554.roms).

Both hardware prefetching and increasing LLC size have the same goal, improving performance by decreasing cache misses. Therefore, when either one of these two techniques acts effectively, it removes part of the problem and reduces the need for the other one. Hardware prefetching is very effective for many applications. Even with the minimum LLC size (1.75 MiB in our system), MPKI3 is very low for many benchmarks running with prefetching activated. In all these cases, increasing the LLC size does not provide any benefit. For instance, 510.parest_r.1 clearly shows this behavior. Without prefetching, MPKI3 decreases from 12.5 to 0.6 by increasing the LLC size from 1.75 to 14 MiB. However, when enabling the prefetchers, the MPKI3 with 1.75 MiB is already 1.0, so any further increase in the LLC size provides very little benefit.

Hardware prefetching is very effective in reducing MPKI3 for *all* LLC sizes in 14 and 10 CPU2006 and CPU2017 benchmarks, respectively. This is because prefetching, regardless of the size of the cache, detects the right patterns and goes ahead of the memory reference stream correctly. Therefore, in those benchmarks where MPKI3 is high for all LLC sizes, hardware prefetching is effective in reducing the MPKI also for all LLC sizes.

It also reduces MPKI3 for *small* LLC sizes in other 4 CPU2006 benchmarks (401.bzip2, 473.astar, 482.sphinx3 and 483.xalancbmk) and 2 CPU2017 ones (510.parest and 523.xalancbmk). For one benchmark, omnetpp, which is included in both suites, LLC misses are not reduced for any LLC size, and even are slightly increased with the largest LLC size.

**Fig 6:**
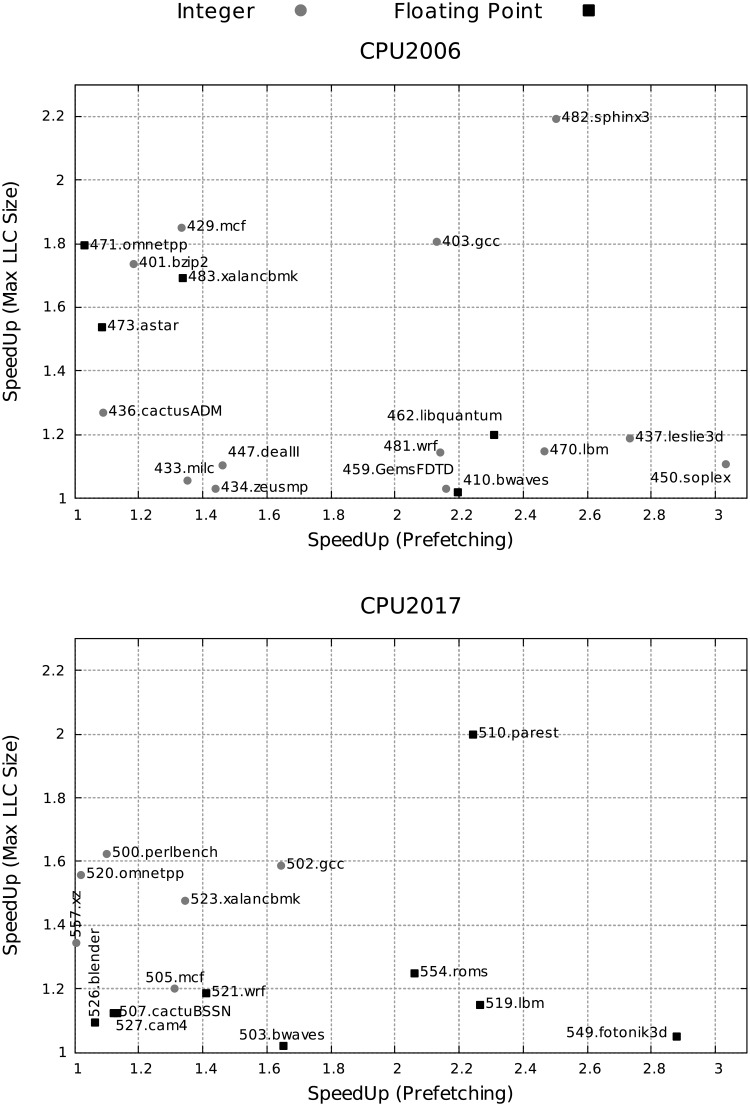
Speed-ups enabled either by hardware prefetching, with the minimum cache size (X axis) or maximum LLC size, without prefetching (Y axis) over a baseline configuration without prefetching and minimum LLC size for the selected SPEC CPU2006 and CPU2017 benchmarks. Integer and floating point benchmarks are represented by gray circles and black squares, respectively.

To summarize, Fig 6 shows the speedups of benchmarks when prefetching is enabled with the minimum cache size (X axis), and when the LLC size is increased up to 19.25 MiB without prefetching (Y axis) with respect to a baseline system with the minimum LLC size and without prefetching. Fig 6 facilitates the classification of benchmarks according to their sensitivity to both parameters. Integer and floating point benchmarks are plotted with gray circles and black squares, respectively. For instance, we can see a group of CPU2006 benchmarks that are very sensitive to hardware prefetching but show little sensitivity to LLC size increase (462.libquantum, 481.wrf, 459.GemsFDTD, 410.bwaves, 470.lbm, 437.leslie3d and 450.soplex).

Fig 6 also allows us to analyze whether the clustering of applications made by other proposals is in agreement with the sensitivity of these applications with respect to the prefetch and the increase in LLC size. As an example, Limaye et al. [18] classify 510.parest and 503.bwaves of SPEC CPU2017 as very similar benchmarks. However, in our classification we can see that 510.parest is the CPU2017 benchmark which is most sensitive to the LLC size while 503.bwaves is the least sensitive one.

#### Correlation between MPKI3 and CPI

**Fig 7:**
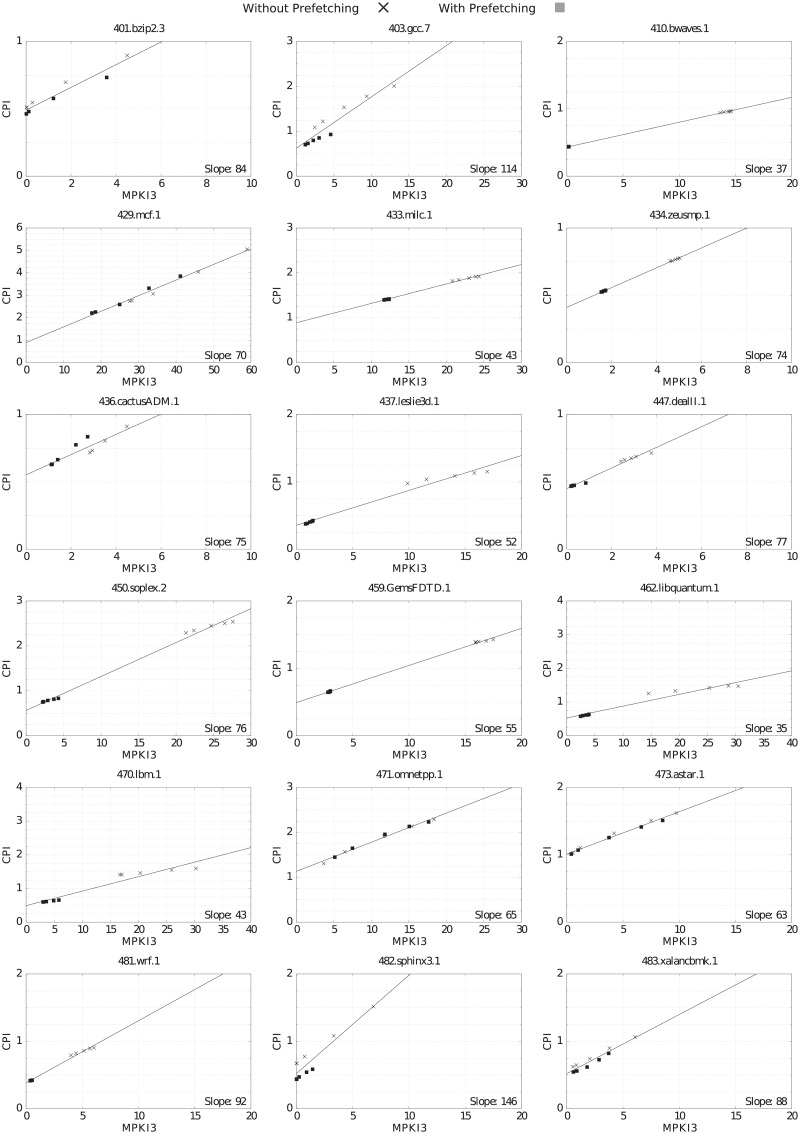
CPI vs. MPKI3 for the selected CPU2006 benchmarks, varying LLC size and with prefetching (square marks) and without prefetching (x marks). Slope units are cycles/miss. Slopes are comparable in all graphs because the ratio between X and Y scales is constant (10:1).

**Fig 8:**
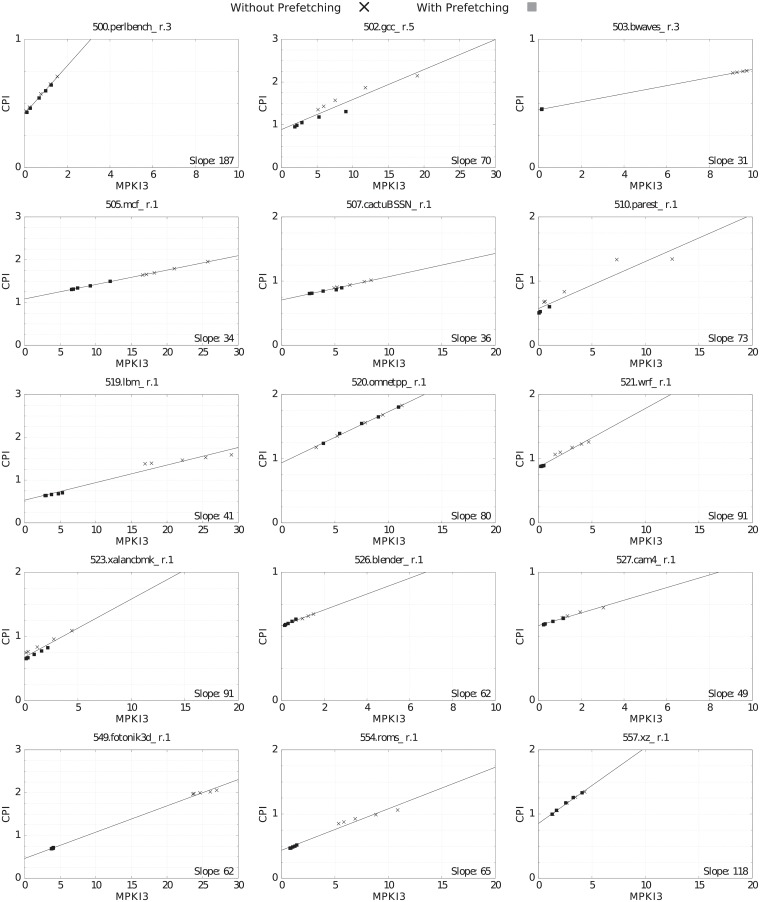
CPI vs. MPKI3 for the selected CPU2017 benchmarks, varying LLC size and with prefetching (square marks) and without prefetching (x marks). Slope units are cycles/miss. Slopes are comparable in all graphs because the ratio between X and Y scales is constant (10:1).

Figs 7 and 8 show the correlation between MPKI3 (X axis) and CPI (Y axis) for the different LLC sizes, with (square marks) and without (x marks) prefetching. The slope of the CPI/MPKI linear interpolation gives thousand of cycles per LLC miss: (cycles/instruction)/(LLC misses/Kinstruction). For instance, the slope of 401.bzip2.3 in Fig 7 is 0.084 Kcycles/LLC_miss. For the sake of clarity, inside the figures we write instead 84 cycles/LLC_miss. This number represents the average LLC miss penalty for the benchmark.

As can be seen in Figs 7 and 8, the linear relationship between MPKI3 and CPI is strong, although the MPKI increase has different impacts on the benchmarks’ CPI. The slope of the interpolation line varies between 30 cycles per miss for several benchmarks in both suites and 187 cycles per miss for 500.perlbench_r.3. The slope value provides another criteria to classify benchmarks according to the amount of instruction-level and memory level-parallelisms [22]. For instance, a large slope value means a low instruction-level and memory-level parallelism (low temporal overlapping among computation and LLC misses, and low temporal overlapping of LLC misses with themselves), as seen in 482.sphinx3.1 and 500.perlbench_r.3.

### Performance of the hardware prefetchers

In this section we analyze the impact of the different hardware prefetchers on the benchmarks’ performance and bandwidth consumption. Intel SKL-SP processors have four hardware prefetchers associated with the first and second cache levels [12]: *L1 Data cache unit prefetcher* (DCUI), *L1 Data cache instruction pointer stride prefetcher* (DCUP), *L2 Data cache spatial prefetcher* (L2A) and *L2 Data cache streamer* (L2P).

**Fig 9:**
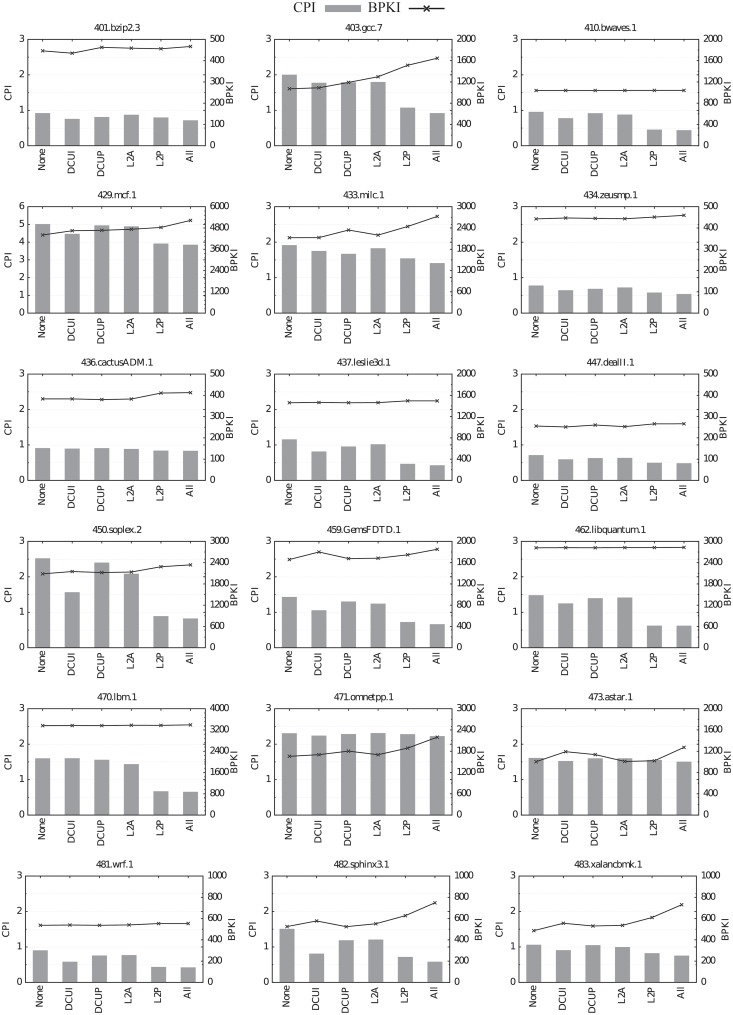
Impact of the different hardware prefetchers on performance (CPI, bars) and bandwidth consumption (BPKI, line) for the selected CPU2006 benchmarks.

**Fig 10:**
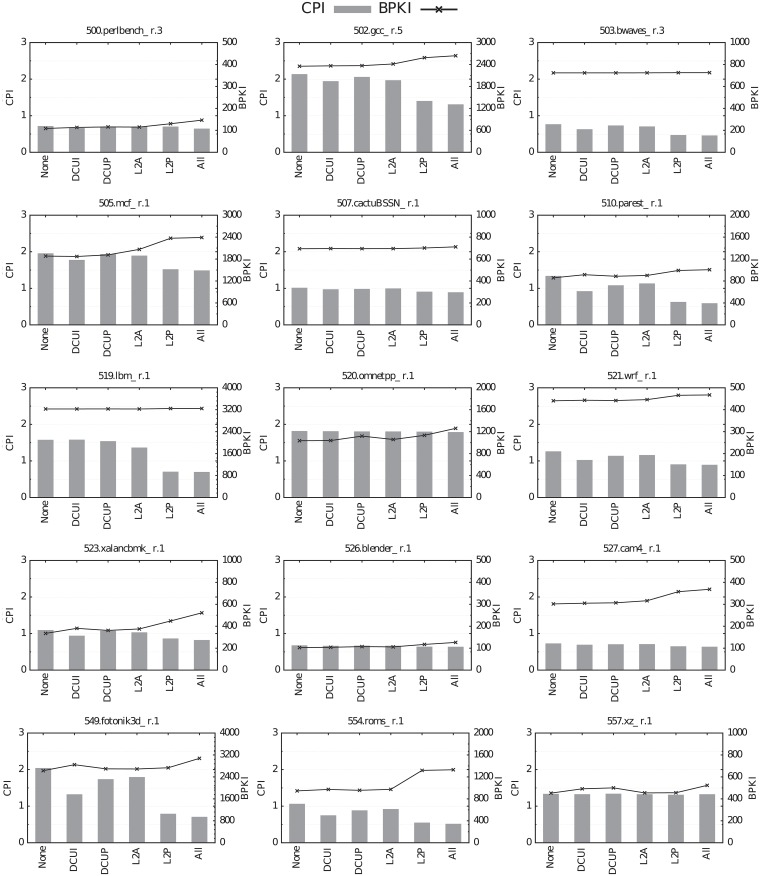
Impact of the different hardware prefetchers on performance (CPI, bars) and bandwidth consumption (BPKI, line) for the selected CPU2017 benchmarks.

All the benchmarks selected in Section *Identification of memory intensive benchmarks* have been executed with different configurations: all prefetchers enabled, all prefetchers disabled and each prefetcher individually enabled. The experiments have been performed with the maximum LLC size. Figs 9 and 10 show performance measured in cycles per instruction (CPI, left axis, bars) and bandwidth consumption measured in bytes read from main memory per kilo instruction (BPKI, right axis, line).

L2P is by far the best prefetcher. For the 14 CPU2006 benchmarks whose miss ratios are reduced by turning on hardware prefetching with the maximum LLC size, L2P, by itself, achieves more than 70% of the CPI reduction obtained with all prefetchers enabled. Furthermore, for 10 of these 14 benchmarks, L2P is responsible for more than 90% of the CPI reduction obtained with all the prefetchers enabled. Similar results are observed for the CPU2017 suite. For the 10 benchmarks that even with the biggest LLC size take advantage of hardware prefetching, L2P alone achieves more than 82% of the CPI reduction obtained when all prefetchers are active. This percentage is greater than 90% for 7 out of these 10 benchmarks.

The second-best prefetcher is DCUI, followed by DCUP. L2A obtains the worst results, since it only reduces CPI in more than 5% for 8 and 6 of the CPU2006 and CPU2017 benchmarks, respectively, with a maximum of 20% for 450.soplex.

Hardware prefetching is very accurate, since it only causes a significant increase of bandwidth consumption in 3 benchmarks of the CPU2006 suite (403.gcc.7, 433.milc and 471.omnetpp) and in 3 CPU2017 benchmarks (520.omnetpp, 549.fotonik3d and 554.roms). Moreover, in most of these benchmarks prefetching causes a considerable CPI reduction despite the increase in bandwidth consumption, with the exception of omnetpp (both 471.omnetpp and 520.omnetpp).

### Temporal evolution of the benchmarks

**Fig 11:**
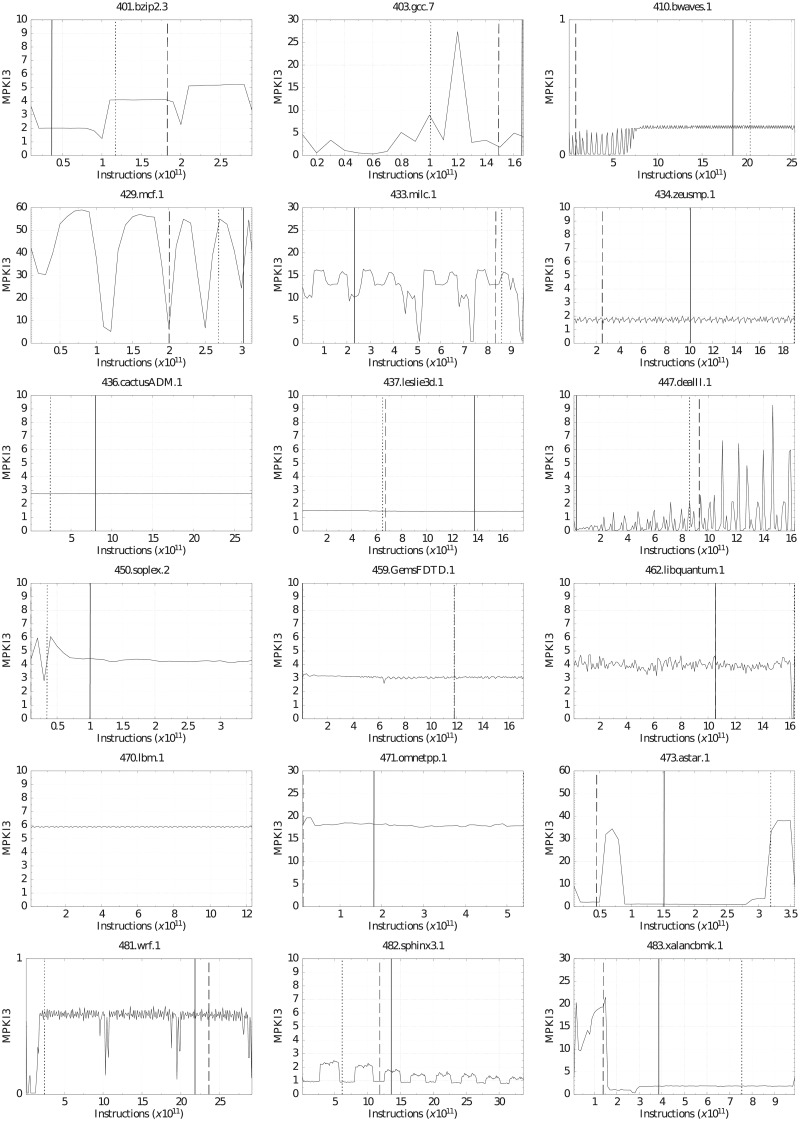
Temporal evolution of MPKI3 and *SimPoint* selection for the selected CPU2006 benchmarks, with minimum LLC size and hardware prefetching.

**Fig 12:**
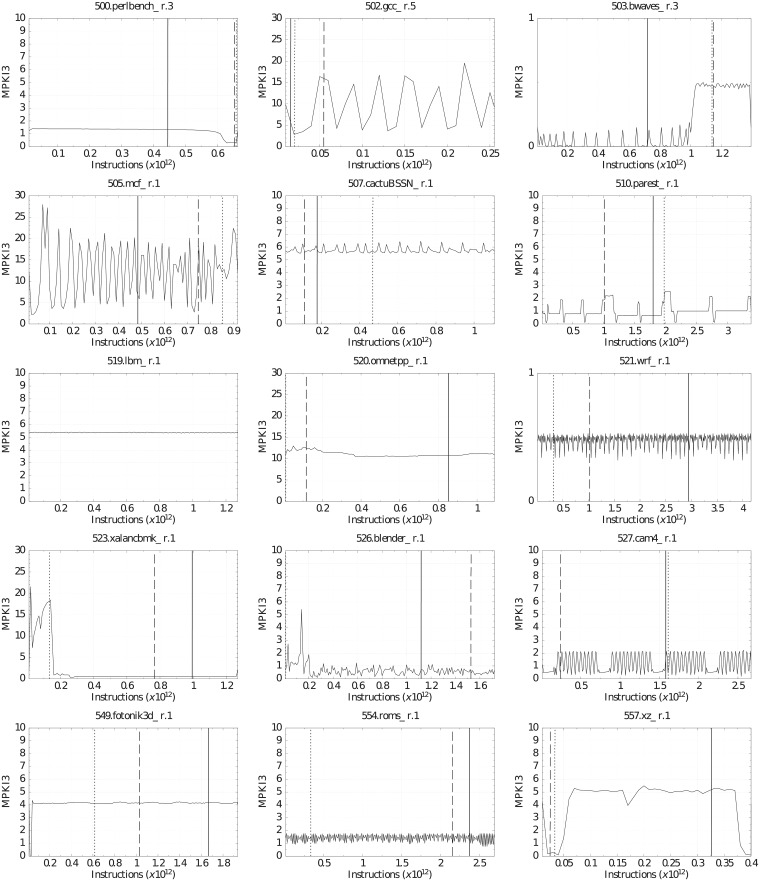
Temporal evolution of MPKI3 and *SimPoint* selection for the selected CPU2017 benchmarks, with minimum LLC size and hardware prefetching.

This section analyzes the temporal evolution of the benchmarks. Figs 11 and 12 show MPKI3 (Y axis) for every million of executed instructions (X axis). This allows us to know the different phases of a benchmark and can help select simulation intervals. The execution was performed with the minimum LLC size (1.75 MiB) and with all the hardware prefetchers enabled. We did the same analysis using other LLC configurations and even using the metrics of the other cache levels (MPKI2 and MPKI1), and a total similarity was observed in all experiments. Thus, the temporal evolution of the applications seems to be very independent of the LLC configuration.

The graphs in Figs 11 and 12 also plot three vertical lines of different patterns representing the first three simulation intervals obtained by *SimPoint* with the parameters MaxK = 3 and interval size = 300 million instructions. The solid, dotted, and dashed lines correspond to the most, second most, and third most representative intervals, respectively. The thickness of each vertical line is not proportional to the number of instructions that it represents. It has been increased in order to improve visibility.

The simulation intervals selected by *SimPoint* are not always representative of all the different phases of a benchmark. As an example, we can observe that 401.bzip2 in Fig 11 clearly has three phases with different MPKI3 values and similar duration, approximately 100 million instructions each one. The MPKI3 of each phase is very different with values around 2.0, 4.1 and 5.2, respectively. However, SimPoint selects its first interval from the first phase, the next two intervals from the second phase, and does not select any interval from the third phase. This is because Simpoint, as the methodology used to select applications, focuses on memory unrelated parameters, giving accurate outcomes, but only to evaluate design tradeoffs related to those parameters.

This analysis reveals the limitations of *SimPoint* to obtain representative intervals of a benchmark execution from the memory hierarchy point of view. The problem gets worse because most research papers based on this methodology only select the first interval.

## Conclusions

In this paper we have analyzed the performance of the memory hierarchy of an Intel Xeon Skylake-SP processor executing the SPEC CPU2006 benchmarks and CPU2017 single-threaded benchmarks. Below, we summarize the main conclusions that we can draw from this characterization.

A significant number of the benchmarks have very low miss ratios in the second and third level caches, even with a small LLC size and without hardware prefetching. The CPU2017 demand for memory hierarchy resources is lower than the CPU2006 one.

We offer a classification of the benchmarks that demand resources in LLC according to their sensitivity to LLC size and hardware prefetching. Hardware prefetching is very effective in reducing LLC misses for most benchmarks, even with the smallest LLC size. Increasing the LLC size is also effective in reducing LLC miss counts for many benchmarks.

The best prefetcher implemented in the SKL-SP processor is L2P. For most benchmarks, it is responsible for 90% of the CPI reduction when using prefetching.

Hardware prefetching is very accurate. In general, the number of bytes read from main memory hardly increases.

Our analysis shows that the methodologies used in other works to select benchmarks [18] and simulation points [7] do not guarantee that representative workloads from the memory hierarchy point of view are obtained.
