# Correction: Memory hierarchy characterization of SPEC CPU2006 and SPEC CPU2017 on the Intel Xeon Skylake-SP

**DOI:** 10.1371/journal.pone.0303712

**Published:** 2024-05-09

**Authors:** Agustín Navarro-Torres, Jesús Alastruey-Benedé, Pablo Ibáñez-Marín, Víctor Viñals-Yúfera

The Data Availability statement for this paper is incorrect. The correct statement is: All data are available at https://github.com/agusnt/Xeon-SP_Memory_Characterization_SPEC-CPU-2K6-2K17/.
